# Medical and Physical Intelligence

**Published:** 1801-04

**Authors:** 


### MEDICAL AND PHYSICAL INTELLIGENCE.

### [FOREIGN AND DOMESTIC.]

### *Extract of a paper on the genus of Lime-tree (Tilia), read at the National Inftitute, by* VENTENAT.

LINNÆUS has mentioned in his Species Plantarum, only two fpecies of lime-tree, to which he gave the name of *Europaea* and *Americana*; but as there are more diftinct fpecies of this tree to be met with in Europe as well as in America, thofe names cannot conveniently be retained, becaufe one might fuppofe by them, that the other fpecies do not grow in Europe or in America.

Miller, in his Gardener's Dictionary, defcribes two American fpecies of lime-tree, retaining the name of *Americana*, for that which was known to Linnæus, and affigning to the other, brought from Carolina by Catefby, the name of Caroliniana; but this cannot likewife be adopted, fince *Michawy* and other botanifts have difcovered in Carolina, another fpecies of lime-tree, different from that found by Catefby. Mr. Aiton, in his Hortus Kewenfis, has probably changed, on that account, the name of *Caroliniana* into that of pubefcens. Mr. Walther diftinguifhes a fpecies of lime-tree, native of Carolina, "*Tilia Ambricana, floribus nectario inftructis, ftipulis floriferis*;" but as this defcription agrees evidently with all the lime-trees that may grow in the whole extent of America, it is obvious how difficult it would be to determine which fpecies Walther may have meant, if the naturalifts who travelled in Carolina had not brought with them fpecimens of the tree, which the Englifh botanift meant to characterife.

After having given the generic character, *Mr. Ventenat* defcribes the following fpecies, of which he has given figures.

*Lime-trees of Europe, with naked petals.*

*Tilia microphylla.*—Foliis cordato-fubrotundis, acuminatis, argute ferratis, capfula fubglobofa, minime coftulata, tenuiffima, fragili. Var. fructo oblongo, utrinque acuminato, Tilli Hort. Pifan. T. 49. fig. 3.

*T. platyphyllos.*—Foliis cordato-fubrotundis, acuminatis, inaequaliter liter ferratis, capfula turbinata, coftis prominentibus, infignita, lignofa, craffa. Var. Carolina, Ait. Hort. Kew.

*Lime-trees of America; petals provided with a fcale, at their bafis.*

*T. glabra.*—Foliis profunde cordatis, ferratis, glabris; petalis apice truncatis, crenatis, capfula ovata, fubcoftata (T. Americana L.)

Habitat in Virginia et Canada.

*T. pubefcens. Ait.*—Foliis bafi truncatis et obliquis denticulato ferratis, fubtus pubefcentibus, petalis emarginatis, capfula globofa lævi.—T. Caroliniana Mill.

Habitat in Carolina.

Var. leptophylla, foliis tenuiffimis, fubpapyraceis. Habitat in Louifiana.

*T. rotundifolio.*—Foliis cordato-fubrotundis, fubfinuatis, dentatis verticalibus fubtus tomentofis; capfula ovata, obfcuri nervofa. T. alba Ait. argentea Mus. nat.

Habitat in America feptentrionali.

*T. heterophylla.*—Foliis ovatis, argute ferratis, bafi nunc cordatis nunc oblique aut æqualiter truncatis fubtus tomentofis, capfula globofa, multinervofa.

Habitat in Carolina inferiori, et in Marylandia.

Mr. Ventenat terminates this paper, by prefenting fome obfervations on the culture of the fpecies of this genus, on the foil that agrees with them, and on the advantages that may refult from them to the community.

---

### *On a new Method of preparing Ice Vinegar.*

It is about ten years ago that Profeffor Lowitz, of Peterfburgh, difcovered the cryftallizibility of the moft concentrated acetic acid, which, when reduced into that folid ftate, obtains the name of *ice vinegar.* This high degree of concentration is chiefly attained through the medium of fulphuric acid, with the help of a hard froft during the winter: The proportion in which this acid was added to the acetic falt, according to the prefcription of Mr. Weftendorf, is 1 : 2. Profeffor Lowitz, however, propofes an eafier and more profitable method for exhibiting from wine vinegar, as well as from that of malt liquors, an ice vinegar, which is much fuperior in ftrength to that prepared in the former way. By taking to *three* parts of the acetic falt *four* parts of fulphuric acid, from a hundred parts of the falt, fixty-one parts of ice vinegar will be obtained, which, when once coagulated, requires 10° Reaumur for being again liquefied. But the whole proceeding is as follows: Saturate three or four pounds of purified potafh with wine or beer vinegar, which has been diftilled over charcoal powder; evaporate the faturated liquor to the confiftence of a dry powder, of which put three pounds, accurately weighed, when ftill warm, into a glafs, previoufly heated, and fhut it with a glafs ftopper. Then pour three pounds of fulphuric acid into a retort, provided on its upper part with a pipe, and join to it a receiver, large enough enough for containing about twenty pints of water. Begin to add to the fulphuric acid the above falt in fmall portions, fhaking and ftirring it frequently. After having mixed all the falt, add by degrees one pound more of fulphuric acid, and fhut the pipe with a wet bladder; fuffer the whole to ftand quietly one night. The next morning place the retort into the fand pot of a furnace, fo deeply, that the fand between the bottom of the pot and the retort be only about half an inch thick; put the receiver into a refrigatory filled with very cold water, after which apply a gentle fire. About one hour after, the diftillation commences by white fumes appearing in the veffels, at which time the fire muft be very carefully managed. The drops that go over may fucceed one another quickly, without any danger of the veffels being cracked; but be very careful that no coherent ftreams run over, and likewife take care that the thick and white fumes only lodge in the loweft part of the receiver; and when they begin to rife, particularly with a whirling motion, take the fire immediately out of the furnace. It is, befides, neceffary to refrigerate often the upper part of the receiver with cold water, or, which is ftill better, with fnow or ice. —The ending of the diftillation is known by the difappearance of the white fumes, by the drops running over much flower, and particularly by the liquefaction of the refiduum to a black frothing fluid that goes eafily over into the receiver. At the moment of the liquefying and frothing of that fubftance, the receiver ought to be taken off, and another put on, into which five or fix drachms of a much weaker and difagreeably fmelling acetous acid will go over; that, however, may be ufed for purifying the ice vinegar from the adherent fulphureous acid; when, after being diluted with water, it is faturated with barytes, filtrated, and evaporated to drynefs. The refiduum is ground to a fine powder, and together with charcoal powder, added to the ice vinegar; after which the mixture ought to be rectified over a gentle fire to the drynefs of the refiduum. Of 3lb. of acetite of kali, 22 oz. of ice vinegar were obtained by this new method. *(Crell's Chemical Annals,* 1800.)

---

*On the internal ufe of Phofphorus.*— Prof. Brera relates a cafe, where two grains of phofphorus triturated with fweet oil and the yolk of an egg, and mixed with cinnamon water, brought on death, by caufing burns in the inteftinal canal, though they were only given by degrees, in the fpace of two days; and befides, an experiment with a dog of eight months, that was killed after five days, by four grains of phofphorus triturated with gum arabic, and adminiftered in four dofes in the courfe of one day. A fimilar inftance is mentioned by Dr. Kortum, of a man, who having fuffered an apoplectic fit, retained a palfy of the left arm, and a weaknefs in the left foot, againft which, all poffible ftimulants had been employed in vain. At laft the doctor determined to try the phofphorus, and two grains of it were accordingly triturated with gum arabic and fweet almonds, and an emulfio phofphorata prepared after the prefcription of Prof. Hufeland, confifting of feven ounces.

Of

Of this emulſion the patient was ordered to take only twice a day a table ſpoonful, in order to ſee whether his weak ſtomach could bear the remedy; but four days after, having hardly taken one half of the mixture, and conſequently only one grain of phoſphorus, the patient felt himſelf much heated, the pulſe became febrile, a congeſtion of blood towards the head aroſe; ſeveral bilious vomitings enſued, and the patient complained particularly of a diſagreeable tenſion and dryneſs in the breaſt. Cooling purgatives and an emulſion of ſweet almonds removed thoſe complaints in a few days, though the tenſion in the breaſt, with much coughing, remained a long time after, and the ſtomach became ſo weakened, that it required for ſeveral weeks the uſe of bitters before it was reſtored. It appears from theſe inſtances, how cautiouſly that powerful remedy muſt be uſed. (*Hufeland's Practical Journal.*)

---

For diſtinguiſhing the rheumatic head-ach from that which ariſes from crudities in the ſtomach, Dr. Dürr points out the following diagnoſtics: 1. The firſt remits ſometimes ſo much of its vehemence, that the patient remains free from it for ſeveral hours, whereas the other kind of head-ach continues with equal violence. 2. On moving the head the patient feels greater pains in the rheumatic head-ach. 3. This kind of head-ach is increaſed by cold, and the patients are therefore obliged to cover the head. 4. As ſoon as pains come on in other parts of the body, it begins to abate, and increaſes again when thoſe go off. 5. The leaſt motion of the head excites that head-ach again, after its having previouſly abated. 6. The rheumatic head-ach is limited to one part of the head, whereas that ariſing immediately from the ſtomach affects the whole head, or, at leaſt, the fore and back part of it. (*Ibid.*)

---

Dr. Corradori made ſome time ago the intereſting obſervation, that the *Tremella Noſtoc* had changed into three different plants, viz. *Tremella verrucoſa*, *Lichen faſcicularis*, and *rupeſtris*. He remarked afterwards, that it was likewiſe transformed into *Tremella lichenoides*, or Lichen tremelloides, *L.* though a part kept the form of *Noſtoc*. Growing upon moſſy ſlopes, it changed into *L. gelatinoſus*; upon ſtones or ſand into *L. criſpus*. On moiſt rocks, the *Tremella Noſtoc* was changed into a plant, coming near to *L. rupeſtris*. *Tremella verrucoſa* and *L. criſpus*, which are found on old walls, were metamorphoſed into *L. granuloſus*. Theſe curious changes are particularly favoured by wet and warm weather; but when the Tremella has reached a certain age, it remains conſtant; if it has once changed, this protheiform plant never recovers its former ſtate. Of Tremella, he gives the following definition, "A plaſtic vegetable ſubſtance, which is nouriſhed by intus-ſuſception." (*Annali di Chemica et Storia Naturale*, *T.* 17, 1798, No. 101.)

---

Dr. Corradori has communicated ſeveral intereſting obſervations on *glow worms*, and their difference with reſpect to their colour, and the number of ſhining rings. Several naturaliſts have attempted to explain this phenomenon, amongſt whom *Spalanzani* aſcribed it to a ſlow combuſtion; but this Dr. Corradori denies, and rather thinks it owing to a diſſipation of aggregated light, an opinion which ſeems to be confirmed by his experiments with worms put in oil, or boiling water. He proves it likewiſe by the experiments of *Becherheim*, who has ſhewn that theſe worms thrown into gaſes improper for combuſtion, continued to give light. Beſides this, *Beccari*, *Brugnatelli*, and *Dufay* have demonſtrated, that light is capable of combining and aggregating itſelf in bodies in more or leſs quantity, in proportion to their capacity of receiving it. He next proceeds to the queſtion, whether theſe ſhining worms are chryſalides or female individuals of different winged inſects, to which latter opinion, adopted by Linnæus, he is inclined to aſſent. According to which, *Lampyris ſplendidula* is a ſpecies of phoſphoric inſect, the female of which is not winged, and produces that light. The blackiſh glow-worm ſeems to be a different ſpecies of Lampyris, and not Italica, for which it was taken by Linnæus. (*Ibid.*)

---

The following is the arcanum againſt the tape worm, for which the late Mr. Mathieu, apothecary, at Berlin, received an annual penſion from the preſent King of Pruſſia.

℞. *Limaturae ſtanni Angl. pur. unc. j. pulv. radic. filicis maris dr. vj. pulv. ſemin. Cynae unc. dimidiam, pulv. radic. Jalappae reſinoſae, Salis polychreſti ana dr. j. Miſc. fiat cum mellis communis ſufficiente quantitate electuarium.*

℞. *Pulver. rad. Jalapp. reſinos. Salis polychreſt. ana ſcrupul.* xij. *Scammonei. Aleppenſis ſcrupul.* j. *Gummi guttae gran.* x. *Miſce fiat cum melle communi electuarium.*

1. In employing this remedy, it is neceſſary for the patient to keep, ſeveral days before, a frugal diet, and not to eat any ſalt meat, &c. but only broth and vegetables. 2. For the cure, a tea ſpoonful of the electuary is taken every two hours, which muſt be continued for two or three days, till the patient feels a ſenſation of the worm in the bowels. 3. He is now to take of the purgative electuary, likewiſe every two hours, a tea ſpoonful, till the worm goes off. If this ſhould not take place, ſome Caſtor oil may be given with it, or applied in clyſters. 4. Age, ſex, conſtitution of the patient, make an important difference in the application of this remedy, and its doſe, and it is therefore proper to try it under the care of an experienced phyſician. Finally, it remains to be obſerved, that the efficacy of this remedy depends in a great meaſure on the root of the fern, and particular care ought therefore to be taken, to employ the root of polypodium filix mas, which, when reduced to powder, is of a reddiſh colour.—(*Salzburg Medical Chirurgical Gazette*, in German, No. 39.)

---

A certain Dr. Lehnhardt, invented ſome time ago a ſalutary liquor for pregnant women, which he ſold as an arcanum, and recommended as an infallible remedy to prevent difficult labours. It

It made a very great noife, and was highly praifed by the inventor, in the ftile of a quack; for which purpofe feveral letters, addreffed to the doctor and to the public, were publifhed in fundry German newfpapers, full of gratitude towards the inventor for his excellent remedy, and of praife of the liquor, as a moft important difcovery for the community. By the help of this liquor, women were faid to be fafely delivered, without the application of any inftrument, to which they had in former labours been obliged to have refort; and, according to the reports, which were loudly fpread, of the remedy, Juno Lucina ought to be in future reprefented with a bottle of Dr. Lehnhardt's liquor in her hand. It may be eafily imagined, how favourably fuch a beneficial remedy was received by the fair fex; and, on the other fide, how zealoufly the inventor was attacked by profeffional men.

Of this famous liquor, a chemical analyfis had already been made by a Mr. Thorey, who found it to confift of one ounce and $1\frac{1}{2}$ drachm of Epfom falt, (magnefia vitriolata) of $1\frac{1}{2}$ drachm of Tartarus vitriolatus, and of eight ounces of water. Prof. Manthey of Copenhagen, however, undertook, at the requeft of an eminent practitioner, a new analyfis of it, which is publifhed in Tromsdorf's Journal for Pharmacy, Vol. 8, No. 1, 1800, and differs in fome refpects from the former.—The liquor is fold in white fquare bottles fealed, with a ticket, on which is printed in German, "Dr. Lehnhardt's new invented falubrious liquor for pregnant women." The colour is of a beautiful dark red, like red French wine; the tafte, a faline bitter, but not very difagreeable; the fmell, a little vinous; and at the bottom of the bottle, a mucilaginous dark-coloured precipitation is generally found. The contents of one bottle weigh about 13 ounces $6\frac{1}{2}$ drachms. The refults of the analyfis were, that it confifts of a folution of Epfom and Glauber's falt, that no vitriolated tartar is contained in it, and but a little muriate of magnefia, which frequently is prefent in the Epfom falt; that the colouring parts were extracted by fpiritus vini. The fynthetical experiments confirmed this analyfis, and a proportionable folution of Sal mirabile Glauberi and Epfom falt in water, coloured with a vinous tincture of lignum fandalum, with the addition of fome red wine, exhibited a liquor, which in every refpect refembled that of Dr. Lehnhardt, or in the following prefcription. ℞. *Aquae fontanæ Unc.* vij. *Sal. mirabil. Glauber. Unc. j.* drachms v. *Sal. Epfomens. Unc.* j. *Vin. rubri Unc.* iv.—What efficacy is to be expected from this famous liquor, after the knowledge of its conftituent parts, may be eafily judged.

---

Prof. Abildgaard of Copenhagen, writes in a letter to Prof. Blumenbach, that they have made there many experiments, by injecting medicines into the veins of horfes, by which means, he affures him, he has cured difeafes that are deemed either incurable or very flowly to be cured, and particularly the ftaggers, within three or four days. They ufed for that purpofe an infufion of radix veratri albi, or the root of white hellebore, and it caufed vehement vehement motions in the ſtomach and bowels. In cows alſo, where medicines brought immediately into the ſtomach are of little or no uſe, this remedy proved very ſerviceable, without producing any ill conſequences. An infuſion of ſix grains of arnica, occaſioned in a horſe a perfect lameneſs, which laſted ſeveral hours. (*Voigt's Magazine for Natural Science, etc. Vol. I. No.* 7, 1800, *in German.*)

---

A new edition of the three former works of Dr. JENNER on Cow-pox is in the preſs, to which will be added a fourth, with a plate exhibiting, by means of drawings coloured from Nature, the vaccine puſtule through its progreſſive ſtages of inflammation, veſication, and ſcabbing; the latter will alſo be printed ſeparately, for the conveniency of thoſe who poſſeſs the three former.

---

Dr. MOSSMAN begs leave to inform the Editors of the Medical and Phyſical Journal, that for ſome time paſt he has had many opportunities of inveſtigating the nature of *Idiopathic Fever,* and has devoted much time to an enquiry into the comparative excellence of the different modes of cure. He is extremely diſſatisfied with the curative agents hitherto employed; and is now preparing for the preſs, "A Diſſertation on *Febrile Action,* incluſive of Strictures on the general treatment of *Fevers,* and an exhibition of powers radically and ſpecifically ſubverſive of thoſe morbid phænomena."

---

Mr. JOHN PULLEY, of Bedford, Member of the Royal College of Surgeons in London, intends ſoon to publiſh an Eſſay on the proximate Cauſe of Animal Impregnation; being the ſubſtance of a Paper read and diſcuſſed in the Medical Society at Guy's Hoſpital, in October 1799.

---

The Inſtitution for the Inoculation of the Vaccine Pock is removed from Warwick-ſtreet to a large and more convenient houſe, No. 5, in Golden-ſquare. Subſcribers of One Guinea per annum, are entitled to have two patients conſtantly on the books. Further particulars may be had of the Secretary, at the Inſtitution.

